# Molecular characterization and spoilage potential of yeasts isolated from traditional Egyptian Karish cheese

**DOI:** 10.1186/s40643-026-01116-2

**Published:** 2026-08-17

**Authors:** Reda H. Abdel-Fadeel, Sherif A. Abdella, Tarek M. Abdelghany

**Affiliations:** 1 Dairy Department, Faculty of Agriculture, Al-Azhar University, Cairo, Egypt; 2 Botany and Microbiology Department, Faculty of Science, Al-Azhar University, Cairo, 11725 Egypt

**Keywords:** Karish cheese, Yeast spoilage, *Kodamaea ohmeri*, *Pichia kudriavzevii*, ITS rDNA, Dairy microbiology

## Abstract

Traditional soft cheeses are susceptible to microbial contamination, particularly when unsterilized flavoring additives are used during production or retail handling. This study aimed to quantify and molecularly characterize the yeast flora associated with spiced Karish cheese, a traditional Egyptian fresh cheese supplemented with peppers and olives. Yeast counts ranged from 2.25 to 2.99 log_10_ CFU/g, while molds were detected in 60% of the samples, reaching 1.47 log CFU/g. These counts exceeded the maximum permissible limits specified by the Egyptian Standard (ES 1008-4/2005), indicating potential deficiencies in hygienic practices during production, handling, or storage. Morphological and physiological characterization grouped the 12 yeast isolates into four phenotypic profiles. Representative non-*Saccharomyces* isolates were further identified by sequencing the ITS1–5.8 S–ITS2 rDNA region, followed by BLASTN analysis and phylogenetic reconstruction using the Neighbor-Joining method with 1,000 bootstrap replicates. Two representative species were identified as *Kodamaea ohmeri* (AZ EG 3) and *Pichia kudriavzevii* (AZ EG 2). BLASTN analysis showed > 99% sequence identity with representative GenBank sequences, while phylogenetic analysis placed both isolates within well-supported monophyletic clades (bootstrap > 95%), with evolutionary distances of 0.009 and 0.020 substitutions/site, respectively. The representative sequences were deposited in GenBank under accession numbers PX406129 and PX503693. These findings provide baseline molecular data for the surveillance of spoilage yeasts in traditional Egyptian dairy products. Although the role of spice additives was not directly investigated, the results support the need for future studies to evaluate their contribution as potential sources of yeast contamination in artisanal cheese production.

**Graphical abstract:**

**Figure:**
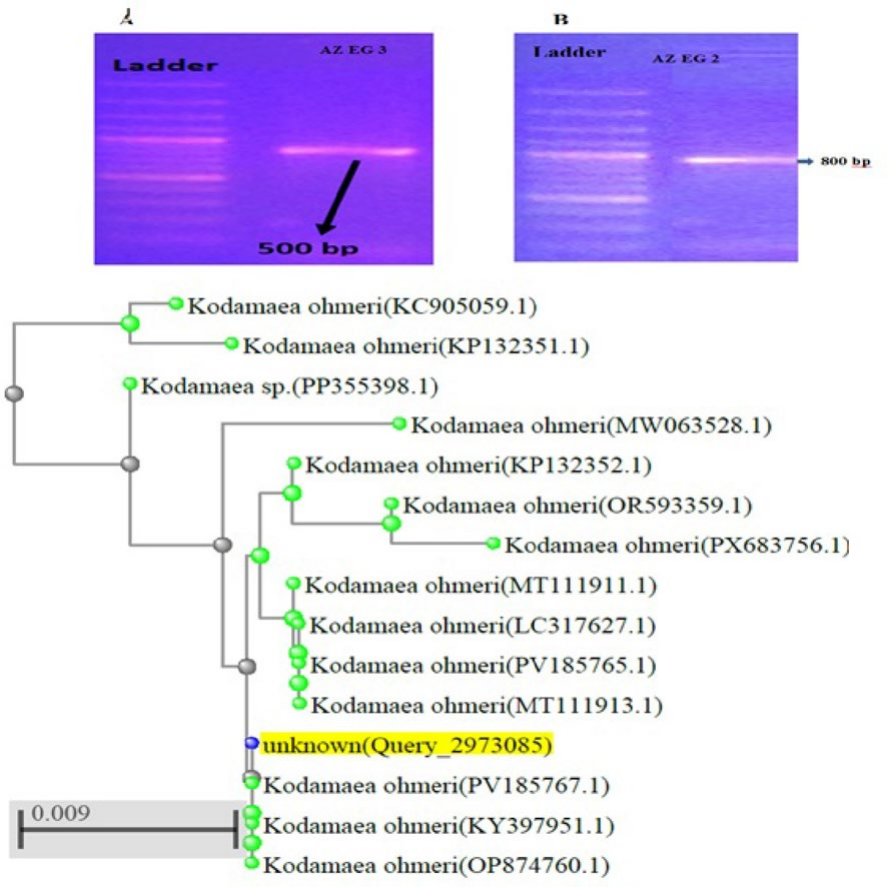

**Supplementary Information:**

The online version contains supplementary material available at 10.1186/s40643-026-01116-2.

## Introduction

The presence of yeast and filamentous fungi in dairy products is frequently undesirable, as they may thrive across a wide range of temperatures and pH levels, leading to severe product deterioration (Moubasher et al. 2018). Their counts serve as an important indicator of hygienic quality and storability. Due to their proteolytic and lipolytic activities, these fungi can produce gas, off-odors, and rancidity in cheese and butter (Snyder et al. 2024). In particular, yeasts from genera such as *Candida*, *Debaryomyces*, *Pichia*, and *Rhodotorula* can metabolize lactose and organic acids, resulting in quality degradation, discoloration, and harsh flavors (Garnier et al. 2017a, b; Geronikou et al. 2020).

Karish cheese, a popular fresh soft cheese variety in Egypt (also known as defatted cheese), is valued for its high protein content and affordable price (Soliman and Aly 2011; Helmy et al. 2019). However, the traditional production process often occurs under unhygienic conditions, providing numerous opportunities for microbial contamination. Additionally, this product is frequently displayed without protective packaging, which substantially increases the risk of contamination by pathogenic and spoilage microorganisms (Todaro et al. 2013; Awad 2016). The low pH, high salt content, and chilled storage conditions of such cheeses favor the development of fungi, including yeasts, as a significant component of the microbiota (Viljoen 2001).

While peppers and olives are commonly used to enhance the flavor of Karish cheese, they serve as more than garnishes; they represent a potential route for the introduction of mycotoxigenic molds (e.g., *Aspergillus flavus* and *Penicillium* spp.) and pathogenic yeasts (e.g., *Candida albicans* and *C. catenulata*) (Soliman and Aly 2011). In traditional Egyptian soft cheeses, unsterilized raw additives can substantially increase fungal loads in the final product, potentially allowing toxin production during storage and posing a public health concern (Moharram et al. 2018).

In the dairy industry, yeasts play dual roles: some contribute to flavor development during ripening, while others serve as biocontrol agents (Psani and Kotzekidou 2006; Sidari and Tofalo 2024). However, several yeast species can metabolize organic acids, raising the pH and promoting the growth of pathogenic bacteria and spoilage organisms (Riesute et al. 2021). Moreover, some yeasts have been linked to opportunistic infections in humans, underscoring the need for accurate identification (Psani and Kotzekidou 2006). Spoilage yeasts are primarily associated with fruity or bitter off-flavors, gas production, and textural defects (Choskit et al. 2023).

In Egypt, the microbiological quality of Karish cheese is regulated by the Egyptian Standard ES 1008-4/2005, which establishes maximum permissible limits for yeast (≤ 400 CFU/g) and mold (≤ 10 CFU/g). Despite these regulatory limits, frequent non-compliance has been reported in artisanal and street-vended products, reflecting persistent contamination challenges (Soliman and Aly 2011). Among emerging spoilage yeasts, *Kodamaea ohmeri* and *Pichia kudriavzevii* have gained increasing attention due to their strong proteolytic and lipolytic activities, thermotolerance, and ability to produce off-flavors, gas, and textural defects in dairy products (Garnier et al. 2017a, b; Srimahaeak et al. 2022; Zhao et al. 2025). Both species are also documented as opportunistic pathogens with antifungal resistance, reinforcing the critical need for accurate molecular identification in food quality surveillance programs (Al-Sweih et al. 2011; Pfaller and Diekema 2007; Kovács et al. 2025).

Despite the widespread popularity of spiced Karish cheese in Egypt, the risk of fungal spoilage and potential exposure to opportunistic species remains a concern due to increasing consumer demand. However, no comprehensive molecular characterization of the associated yeasts has been conducted, limiting the development of effective control strategies and the accurate assessment of health risks. Furthermore, the role of additives such as olives and peppers as potential carriers of opportunistic pathogens and spoilage yeasts remains to be elucidated.

We hypothesized that non-Saccharomyces spoilage yeasts, including opportunistic species, are primarily introduced through unsterilized spice additives (peppers, olives, and spices) used in traditional Karish cheese production, and that fungal loads would exceed Egyptian regulatory limits, indicating insufficient quality control during manufacturing or storage. Therefore, the specific objectives of this study were: (1) to quantify the total yeast and mold populations in spiced Karish cheese samples collected from different production batches obtained from local markets in Giza Governorate; and (2) to isolate and comprehensively characterize the recovered non-Saccharomyces yeast isolates using morphological, physiological, and molecular approaches, including ITS1-5.8 S-ITS2-LSU rDNA sequencing, for accurate species-level identification and phylogenetic confirmation, followed by deposition of representative strains in GenBank.

## Materials and methods

### Samples

Five Karish cheese samples were obtained from city markets, Giza Governorate, Egypt, over a period extending from November 2024 to January 2025. To capture the variability inherent in traditional artisanal production and avoid pseudoreplication, these samples were deliberately collected from different production batches for each vendor and on separate dates. All samples were aseptically transferred into sterile polyethylene bags, placed immediately in an insulated ice box maintained at 4–6 °C, transported to the laboratory within 2–4 h of collection, and processed immediately upon arrival to preserve the original microbiological profile. For microbiological analysis, 5 g of each cheese sample was mixed with 45 mL of peptone water and homogenized. The mixture was then streaked onto Yeast Mold (YM) agar medium (Thermo Fisher Scientific, USA), and the plates were incubated for 48 h at 25 °C. Microbiological enumeration was performed in triplicate for each sample, with the final values presented in Table 1 representing the mean log_10_ CFU/g calculated from the three replicates.

**Table 1:** Fungi count in studied Karish cheese samples

Samples	Microbiological examination		Sources	Number of isolates
	Yeast (log cfu g^−1^)	Mold (log cfu g^−1^)		
*Kc1	2.99	1	Kafr Hakim city at Giza governorate	2
Kc2	2.80	0		3 2
Kc3	2.55	1. 47		2
Kc4	2.25	0		3
Kc5	2.76	1.3		2

*Kc = Karish cheese

### Characterization of morphology and physiology

In order to assess colony morphology (surface, color, margin, and elevation) and microscopic cell appearance, yeast isolates from fresh cultures were cultivated on YPGA agar in Petri dishes (Kurtzman and Fell 1998). Additionally, cultural traits in liquid YPG broth (pellicle, sediment, or ring development) were evaluated by cultivating fresh isolates in YPG broth tubes. Physiological tests included growth under high osmotic stress (50–60% glucose or 5–10% NaCl) (Wickerham 1951), nitrogen assimilation in YCB broth (Suh et al. 2006), starch formation detected with diluted Lugol’s iodine, tolerance to 1% acetic acid (Yarrow, 1984; Suh et al. 2006), and gelatin liquefaction by incubating 2.5 mL inoculated gelatin medium at 25 °C for up to three weeks with periodic checks .Growth was scored semi-quantitatively: (+++) strong, (++) moderate, (+) weak, and (−) none. Observations were recorded at 48 h, 72 h, and 7 days (25 °C). Tolerance was assessed by comparing growth to unstressed controls.

### Molecular identification

Genomic DNA was extracted from pure fungal cultures using the GeneJET Genomic DNA Purification Kit (Cat. No. K0721, Thermo Fisher Scientific, USA) following the manufacturer’s instructions. DNA quality and concentration were assessed using a spectrophotometer (Milton Roy 310, Tokyo, Japan), where purity was confirmed by an A260/A280 ratio of ~ 1.8.

The ITS region (including the 5.8 S rRNA gene) was amplified via PCR using the universal primers ITS1 (5′–TCCGTAGGTGAACCTGCGG–3′) and ITS4 (5′–TCCTCCGCTTATTGATATGC–3′). The PCR reaction was performed in a total volume of 25 µL, containing 12.5 µL of GoTaq^®^ Green Master Mix (Promega, USA), 0.5 µM of each primer, and 100 ng of template DNA. Amplification was carried out in a thermal cycler (Eppendorf Mastercycler Gradient, Germany) with the following profile: initial denaturation at 94 °C for 5 min; 35 cycles of denaturation at 94 °C for 30 s, annealing at 55 °C for 1 min, and extension at 72 °C for 45 s; followed by a final extension at 72 °C for 4 min. PCR amplification was performed by an accredited commercial facility (Sigma-Aldrich) following their standard quality-controlled protocols, which include appropriate controls.

PCR products were verified by electrophoresis on a 1.5% agarose gel stained with ethidium bromide and visualized under UV light. Amplicons were purified using the GeneJET PCR Purification Kit (Cat. No. K0701, Thermo Fisher Scientific, Lithuania) and sequenced bidirectionally by GATC Biotech (Konstanz, Germany) using an ABI 3730xl DNA Sequencer. The obtained sequences were analyzed using the NCBI BLAST tool (nr/nt database) for species identification. Representative sequences were deposited in the GenBank database with accession numbers PX406129 (*Kodamaea ohmeri*, 502 bp) and PX503693 (*Pichia kudriavzevii*, 813 bp).

Phylogenetic analysis was conducted using MEGA software (Karimi et al. 2015; Bessadok et al. 2022). The phylogenetic tree was constructed using the Neighbor-Joining (NJ) method with 1000 bootstrap replicates. Evolutionary distances were computed using the Kimura 2-parameter (K2P) model, which is standard for fungal ITS analysis to account for transitional and transversional substitutions. Gaps were treated as missing data (pairwise deletion). No outgroup was included, as the objective was to confirm species-level identification by clustering query sequences with reference strains rather than to infer deep evolutionary relationships.

## Results and discussion

### Yeast and mold count in Karish cheeses

The mycological examination of Karish cheese samples from Kafr Hakim City, Giza Governorate, is presented in Table 1. Yeast counts varied from 2.25 to 2.99 log_10_ CFU/g, with sample Kc1 having the highest count (2.99) and sample Kc4 having the lowest (2.25). The recorded yeast counts (2.25–2.99 log_10_ CFU/g) align with Elbassiony et al. (2021) for traditional Karish cheese but exceed those reported for plain soft white cheese (Hegazy and Mahgoub 2013). This elevation is attributed to unsterilized spice additives (peppers and olives) acting as contamination vectors in this novel spiced variant. Spices such as peppers and olives are raw agricultural commodities that naturally harbor diverse microbial communities, with typical loads of 10^5^–10^6^ CFU/g (Bedada et al. 2018). Primary contamination sources include epiphytic microbiota, soil contact, airborne deposition, and inadequate drying or ambient storage conditions. In traditional Egyptian dairy production, these additives are incorporated post-processing without prior heat treatment or sterilization, providing a direct route for microbial introduction. This mechanism aligns with previous reports linking unsterilized raw additives to elevated fungal loads in Egyptian soft cheeses (Soliman and Aly 2011; Moharram et al. 2018). Additionally, the identified *K. ohmeri* and *P. kudriavzevii* match spoilage profiles in Egyptian dairy (Soliman and Aly 2011; Moharram et al. 2018); however, this study provides the first molecular confirmation of their prevalence specifically within this emerging spiced product.

Mold contamination was detected in 60% of the tested samples (3/5), with counts ranging from 0 to 1.47 log_10_ CFU/g. Samples Kc2 and Kc4 showed no mold growth, while Kc3 had the highest level (1.47). A moderate level of fungal diversity was indicated by the recovery of two to three fungal isolates per sample. Compliance with Egyptian Standards (ES 1008-4/2005) was assessed by direct comparison, and no inferential statistics were required as this was a compliance evaluation rather than a comparative study. The measured yeast and mold counts exceeded the Egyptian Standards limits for Karish cheese, indicating possible contamination during handling, manufacture, or storage procedures (Hegazy and Mahgoub 2013). Because fungal contamination can result in product degradation and possible mycotoxin development, this raises serious quality and safety concerns and emphasizes the need for better sanitary standards across the cheese production chain.

### Isolation and identification of some yeast spp

The objective of this section was to isolate and characterize non-Saccharomyces yeasts from spiced Karish cheese obtained from local markets in Giza Governorate. Yeast colonies were initially isolated on YM agar medium, and a total of twelve pure isolates were recovered from the five samples. For further characterization, the isolates were maintained in YPG broth and incubated at 25 °C for 48–72 h.

### Morphological and physiological characterization of the tested cultures

Based on identical physiological profiles within each phenotypic cluster, and aligning with the study’s focus on non-*Saccharomyces* spoilage yeasts, representative isolates from the two non-*Saccharomyces* clusters (Patterns 1 and 2) were selected for molecular confirmation. The remaining clusters (Patterns 3 and 4) were confidently identified as *Saccharomyces cerevisiae* and *Kluyveromyces lactis*, respectively, based on well-established phenotypic traits.

Morphological examination of the twelve yeast isolates revealed consistent phenotypic features (Table 2). All strains produced white, creamy colonies with entire margins and a smooth texture on solid media (YPGA at 25 °C for 72 h). Microscopic examination showed that cells were oval to ellipsoidal and reproduced by multilateral budding. The absence of pseudohyphae or true hyphae confirmed the asporogenous yeast morphology characteristic of dairy isolates.

**Table 2:** Physiological characterization of isolated cultures from spiced Karish cheese

Isolates	Pattern grouping	No. of isolates	Fermentation test							Assimilation of carbon compounds					Nitrogen compounds			Complementary tests						Species
			Lactose	Glucose	Raffinose	Sucrose	Galactose	Maltose	Starch	propane-1,2-diol	Butane-2.3 diol	Maltose	Galactose	Citrate	DL-Lysine	Ethylamine	Tryptophane	50% Glucose	60% Glucose	5% NaCl	10% NaCl	Hydrolysis of urea	1% acetic acid	
KC 1, 8, 13	1	3	─	+	─	+	─	─	─	─	─	─	─	─	+	+	─	+	+/-	+	+	+	+	*K. ohmeri*
KC 3, 5, 7, 10	2	4	─	+	+	+	─	─	─	─	─	─	─	─	─	─	─	+	─	+	─	─	**─**	*P. kudriavzevii*
KC 4, 6, 9, 12	3	4	─	+	+	+	+	+	─	─	─	+	+	─	─	─	─	+	─	+	─	─	**─**	*S. cerevisiae*
KC 2, 11	4	2	+	+	+	+	+	+	─	─	─	+	+	+	─	─	─	─	─	─	─	─	**─**	*K. lactis*

Legend: (+) positive; (−) negative; tests not shown (16% NaCl, Gelatin liquefaction) were negative for all isolates

Physiological characterization of the twelve yeast isolates revealed four distinct metabolic patterns based on carbon source utilization and stress tolerance profiles (Table 2). Pattern 1 (isolates KC 1, 8, 13) was identified as *Kodamaea ohmeri*, demonstrating fermentation of glucose and sucrose, along with notable tolerance to high concentrations of acetic acid (1% v/v) and NaCl (≤ 10%). Pattern 2 (isolates KC 3, 5, 7, 10) was identified as *Pichia kudriavzevii*, exhibiting moderate osmotolerance and assimilation of raffinose, glucose, and sucrose. Pattern 3 (isolates KC 4, 6, 9, 12) was identified as *Saccharomyces cerevisiae*, showing a broad fermentative capacity including glucose, sucrose, raffinose, maltose, and galactose, as well as moderate salt tolerance. Pattern 4 (isolates KC 2, 11) was identified as *Kluyveromyces lactis*, distinguished by efficient lactose fermentation and citrate assimilation, which are key diagnostic features for this species. Nitrogen compound assimilation was predominantly negative across all isolates, supporting phenotypic differentiation. Morphological and physiological data were interpreted according to standardized taxonomic criteria (Barnett et al. 2000; Kurtzman et al. 2011).

Based on identical physiological profiles within each phenotypic cluster and considering the study’s focus on non-*Saccharomyces* spoilage yeasts, representative isolates from the two non-*Saccharomyces* clusters (Patterns 1 and 2) were selected for molecular confirmation. The remaining clusters (Patterns 3 and 4) were confidently identified as *Saccharomyces cerevisiae* and *Kluyveromyces lactis*, respectively, based on well-established phenotypic traits.

### Molecular characterization of the tested cultures

#### Identification of PCR products and visualization

**Fig. 1:**
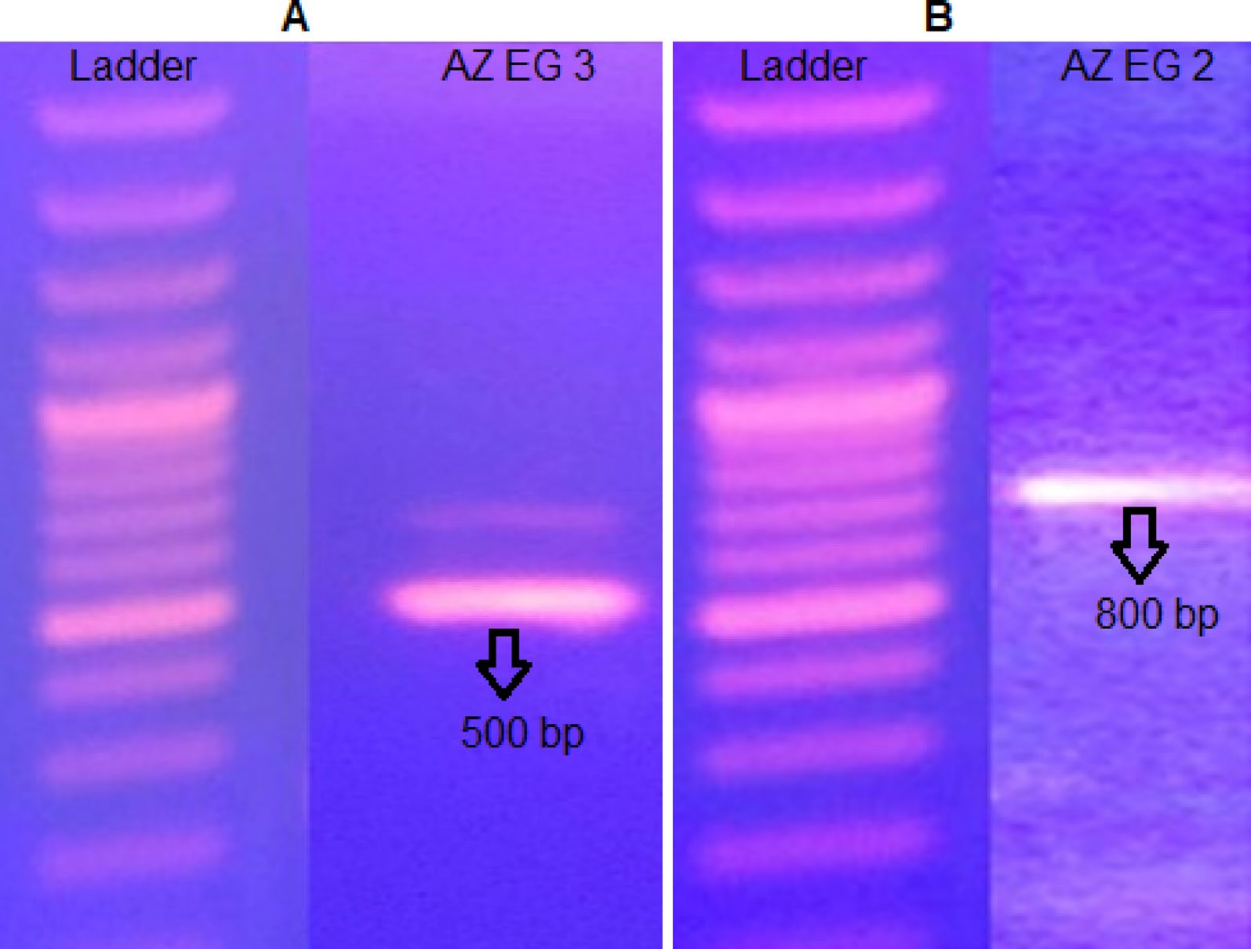
PCR amplification of ITS region using primers ITS1 and ITS4. **A** *K. ohmeri* (strain AZ EG 3 502 bp); **B** *P. kudriavzevii* (strain AZ EG 2* 813 bp)

The PCR amplification results for the ITS region of the two yeast strains are displayed in Fig. 1. Sequence analysis of the partial ITS1, the complete 5.8 S rRNA gene, the ITS2 region, and the partial large subunit (LSU) rRNA gene was used for strain identification. Using primers ITS1 and ITS4, amplification of *Kodamaea ohmeri* produced a product of approximately 502 bp. These results align with the molecular identification frameworks developed for ascomycetous yeasts (Kurtzman and Robnett 1998; White et al. 1990). Additionally, the PCR results for *Pichia kudriavzevii* are shown in Fig. 1B, which displays an amplified fragment of 813 bp targeting the ITS region using the same set of primers (ITS1 and ITS4). Sequence analysis confirmed the identity of both isolates through high similarity matches (> 99%) with reference sequences deposited in the GenBank database.

BLASTN analysis of the ITS sequence of *K. ohmeri* (AZ EG 3) revealed high similarity with representative *K. ohmeri* sequences deposited in GenBank (Supplementary Fig. S1). The closest matches showed 99.39–99.79% sequence identity, 97–98% query coverage, and E-values of 0.0, providing strong molecular support for species-level identification. Representative sequences are listed in Table 3.

**Table 3:** Sequence features, GenBank accession numbers, and BLASTN alignment parameters for the identified yeast isolates

No	Isolate	Species	Length(bp)	Accession No	Locusbp	Query coverage (%)	Identity (%)	E-value	Closest reference strain
1	AZ EG3	*K. ohmeri*	502 bp	PX406129	ITS1-5.8 S-ITS2	97–98	99.39–99.79	0.0	PV185767.1 + OP874760.1
2	AZEG2*	*P. kudriavzevii*	813 bp	PX503693	ITS1-5.8 S-ITS2-LSU	98–99	99.00–99.50	0.0	CP093506.1 + KF959838.1

Sequencing was performed bidirectionally using primers ITS1 and ITS4. Query coverage ranges reflect quality-trimmed sequences following NCBI standard protocols

On the other hand, BLASTN analysis of the ITS sequence of *P. kudriavzevii* (AZ EG 2) revealed high similarity with representative *P. kudriavzevii* sequences deposited in GenBank (Supplementary Fig. S2). The closest matches showed 99.00–99.62% sequence identity, 98–99% query coverage, and E-values of 0.0, providing strong molecular support for species-level identification.  .

#### Database submissions and accession numbers

The representative ITS sequences obtained in this study were deposited in the NCBI GenBank database under accession numbers PX406129 (*K. ohmeri*, AZ EG 3) and PX503693 (*P. kudriavzevii*, AZ EG 2). Sequence characteristics, accession numbers, and BLASTN summary statistics are presented in Table 3.

### Phylogenetic relationship of the Genus *Kodamaea* and *Pichia*

**Fig. 2:**
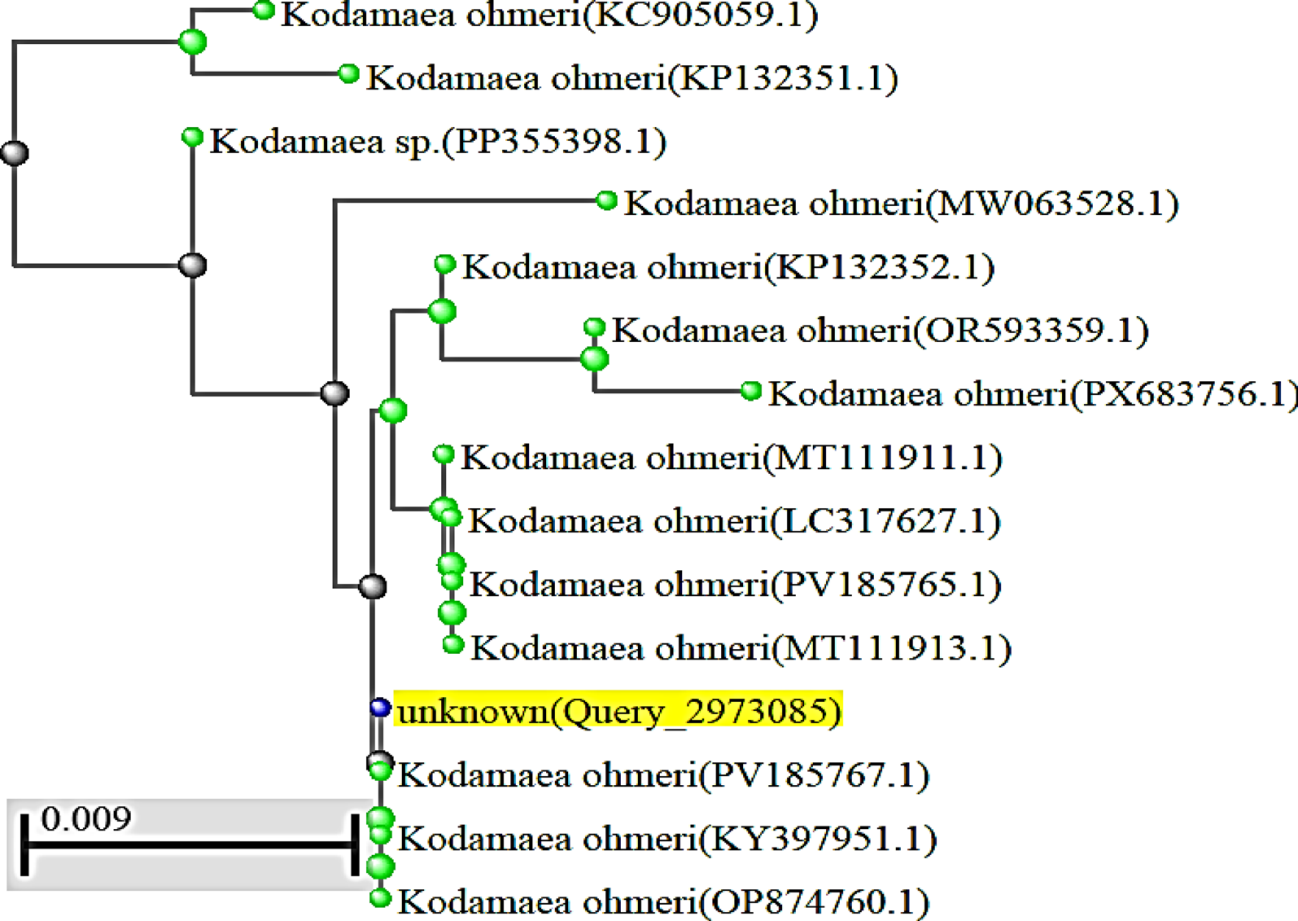
Neighbor-Joining phylogenetic tree based on ITS1-5.8 S-ITS2 rDNA sequences showing the relationship between the Egyptian *K. ohmeri* isolate (AZ EG 3) and representative reference sequences. Bootstrap values (> 50%) based on 1,000 replicates are indicated at the nodes. The scale bar represents 0.07 substitutions per site

**Fig. 3:**
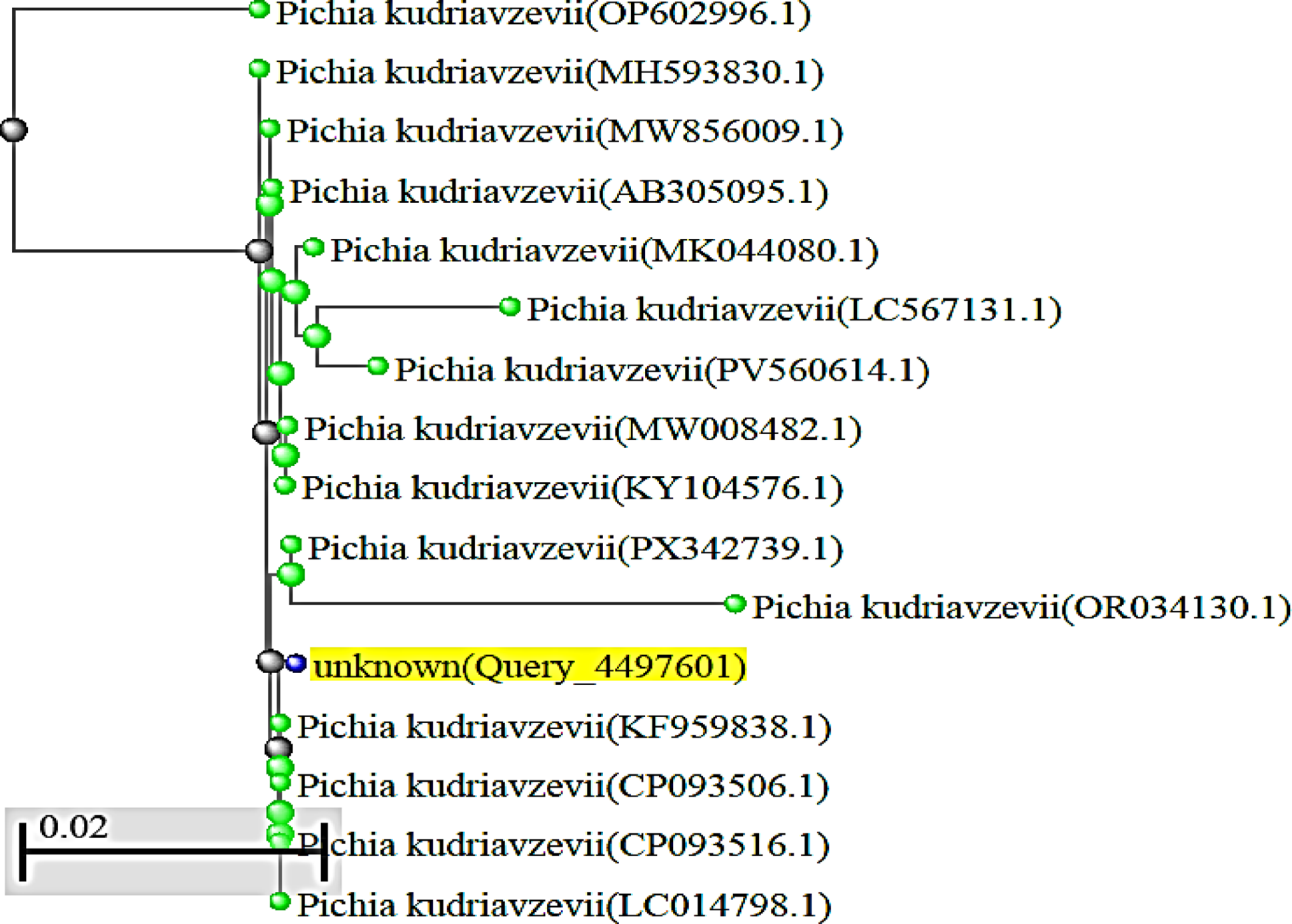
Neighbor-Joining phylogenetic tree based on ITS1-5.8 S-ITS2 rDNA sequences showing the relationship between the Egyptian *P. kudriavzevii* isolate (AZ EG 2) and representative reference sequences. Bootstrap values (> 50%) based on 1,000 replicates are indicated at the nodes. The scale bar represents 0.07 substitutions per site

Phylogenetic reconstruction based on the ITS1–5.8 S–ITS2 rDNA sequences was performed using the Neighbor-Joining (NJ) method with 1000 bootstrap replicates (Figs. 2 and 3). Evolutionary distances were calculated using the Kimura 2-parameter (K2P) model, and gaps were treated as missing data using pairwise deletion.

The isolate *K. ohmeri* (AZ EG 3) clustered with representative reference sequences (PV185767.1, KY397951.1, and OP874760.1) within a well-supported monophyletic clade, exhibiting an evolutionary distance of **0.009 substitutions/site** and bootstrap support exceeding **95%**. Similarly, *Pichia kudriavzevii* isolate AZ EG 2 clustered with representative reference sequences in a distinct monophyletic clade, with an evolutionary distance of **0.020 substitutions/site** and bootstrap support greater than 95%.

The phylogenetic placements were consistent with the BLASTN sequence similarity analysis (Supplementary Figs. S1 and S2), providing strong molecular support for the identification of the isolates as *K. ohmeri* and *P. kudriavzevii*. The representative ITS sequences were deposited in GenBank under accession numbers PX406129 (*K. ohmeri*) and PX503693 (*P. kudriavzevii*.

Both species have been reported as spoilage yeasts in dairy products because of their extracellular proteolytic and lipolytic activities, which may contribute to texture deterioration, rancidity, and undesirable flavor development. In addition, both species have been described as opportunistic human pathogens with documented antifungal resistance (Al-Sweih et al. 2011; Pfaller and Diekema 2007). It should be noted that the food safety implications discussed here are based on published evidence and theoretical risk assessment, since virulence characterization, toxin production assays, and challenge studies were beyond the scope of the present work. Future studies should experimentally evaluate these risks under conditions relevant to cheese production and storage.

## Conclusion

This study provides baseline molecular data on the yeast microbiota associated with traditional Egyptian spiced Karish cheese. Yeast counts ranging from 2.25 to 2.99 log_10_ CFU/g exceeded the maximum permissible limits established by the Egyptian Standard (ES 1008-4/2005), indicating potential deficiencies in hygienic practices during production, handling, or storage. Molecular characterization based on ITS sequencing identified the representative non-*Saccharomyces* isolates as *K. ohmeri* (GenBank accession no. PX406129) and *P. kudriavzevii* (GenBank accession no. PX503693). BLASTN analysis (> 99% sequence identity) together with phylogenetic reconstruction provided strong molecular support for their species-level identification and established reference sequences for future surveillance of spoilage yeasts in traditional dairy products.

The occurrence of these species highlights the importance of monitoring spoilage yeasts in artisanal dairy products because both have been reported in the literature as organisms capable of causing quality deterioration through proteolytic and lipolytic activities and have also been associated with opportunistic infections in susceptible individuals. However, the food safety implications discussed in this study are based on published evidence, as virulence characterization, toxin production, and antifungal susceptibility testing were beyond the scope of the present investigation.

The findings emphasize the importance of implementing good manufacturing practices (GMP) and hazard analysis and critical control point (HACCP) principles during the production and retail handling of traditional Karish cheese, including appropriate hygienic management of flavoring additives. Because the cheese samples were analyzed as finished commercial products, the present study could not directly determine the original source of yeast contamination. Therefore, future investigations should examine peppers, olives, spices, and other additives individually, together with different production stages, to clarify their contribution to fungal contamination. Broader surveys covering additional production regions and seasons are also recommended to establish a comprehensive understanding of the distribution and epidemiological significance of spoilage yeasts in traditional Egyptian dairy products.

## Supplementary Information

Below is the link to the electronic supplementary material.

Supplementary Material 1

Supplementary Material 2

## Data Availability

All raw results available with all authors upon request.
