# Supplementary material for: Molecular characterization and spoilage potential of yeasts isolated from traditional Egyptian Karish cheese
Source: Bioresour Bioprocess. 2026 Aug 17;13(1):117. doi: 10.1186/s40643-026-01116-2 (PMC13481603; doi:10.1186/s40643-026-01116-2)
Supplement: Supplementary file 1 [file 40643_2026_1116_MOESM2_ESM.docx]

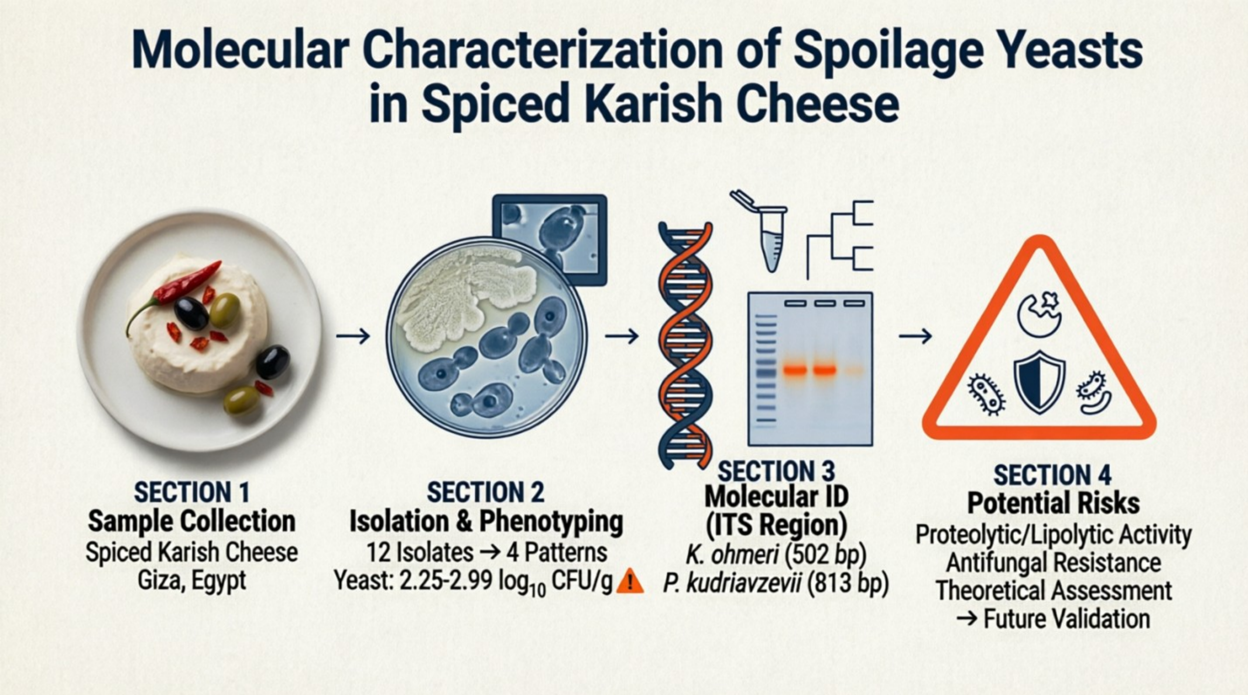
Molecular Characterization of Spoilage Yeasts in Spiced Karish Cheese
SECTION 1
Sample Collection
Spiced Karish Cheese
Giza, Egypt
SECTION 2
Isolation & Phenotyping
12 Isolates → 4 Patterns
Yeast: 2.25-2.99 log_{10} CFU/g
SECTION 3
Molecular ID
(ITS Region)
K. ohmeri (502 bp)
P. kudriavzevii (813 bp)
SECTION 4
Potential Risks
Proteolytic/Lipolytic Activity
Antifungal Resistance
Theoretical Assessment
→ Future Validation

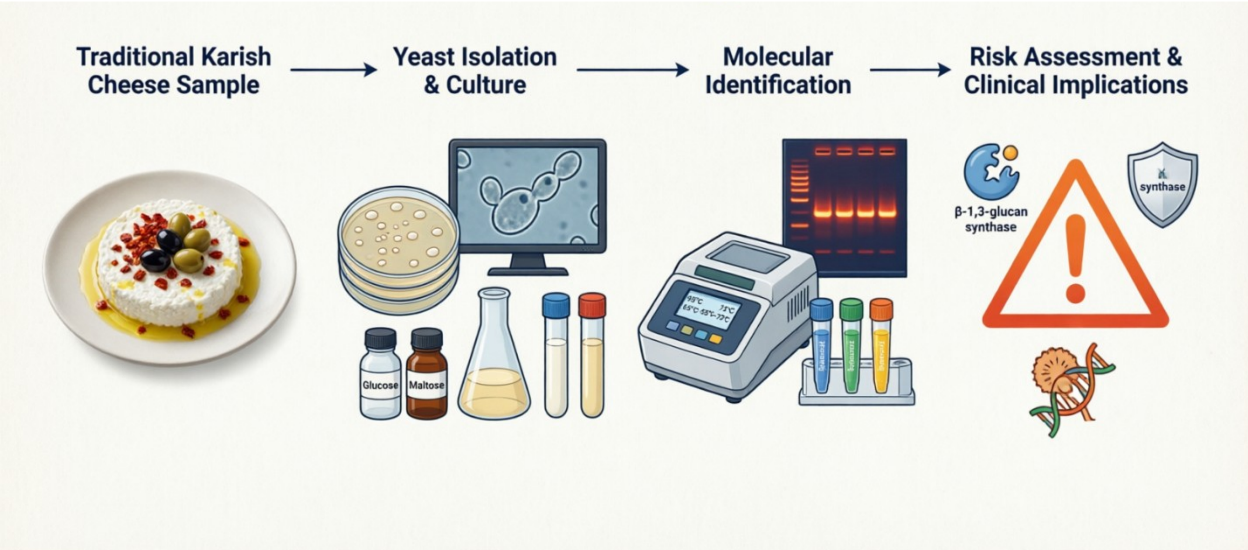
Traditional Karish Cheese Sample
Yeast Isolation & Culture
Molecular Identification
Risk Assessment & Clinical Implications
Glucose
Maltose
β-1,3-glucan synthase
synthase

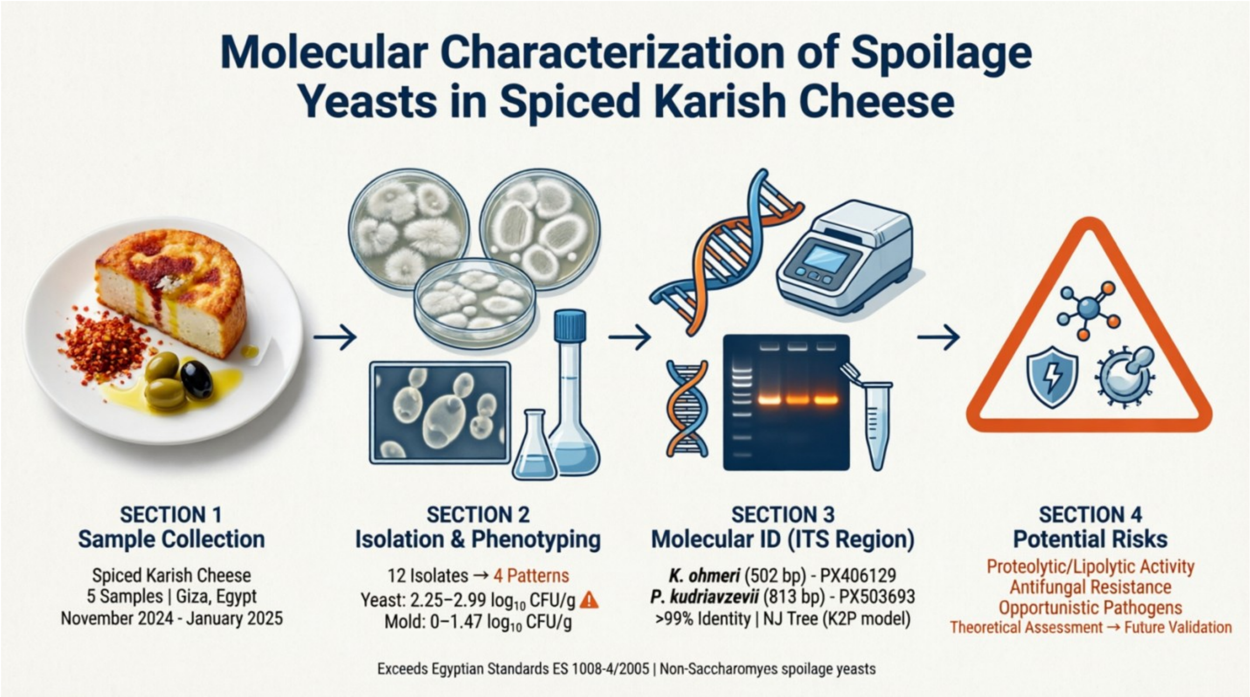
Molecular Characterization of Spoilage Yeasts in Spiced Karish Cheese
SECTION 1
Sample Collection
Spiced Karish Cheese
5 Samples | Giza, Egypt
November 2024 - January 2025
SECTION 2
Isolation & Phenotyping
12 Isolates → 4 Patterns
Yeast: 2.25–2.99 log10 CFU/g
Mold: 0–1.47 log10 CFU/g
SECTION 3
Molecular ID (ITS Region)
K. ohmeri (502 bp) - PX406129
P. kudriavzevii (813 bp) - PX503693
>99% Identity | NJ Tree (K2P model)
SECTION 4
Potential Risks
Proteolytic/Lipolytic Activity
Antifungal Resistance
Opportunistic Pathogens
Theoretical Assessment → Future Validation
Exceeds Egyptian Standards ES 1008-4/2005 | Non-Saccharomyes spoilage yeasts
