# Supplementary material for: Molecular characterization and spoilage potential of yeasts isolated from traditional Egyptian Karish cheese
Source: Bioresour Bioprocess. 2026 Aug 17;13(1):117. doi: 10.1186/s40643-026-01116-2 (PMC13481603; doi:10.1186/s40643-026-01116-2)
Supplement: Supplementary file 1 [file 40643_2026_1116_MOESM1_ESM.docx]

Supplementary Figure S1. Representative BLASTN results for the ITS sequence of *K. ohmeri* isolate AZ EG 3 against the NCBI nucleotide database. The figure shows the top representative BLASTN matches, including GenBank accession numbers, query coverage, percentage identity, and E-values, supporting the molecular identification of the isolate.

Supplementary Figure S2. Representative BLASTN results for the ITS sequence of *Pichia kudriavzevii* isolate AZ EG 2 against the NCBI nucleotide database. The figure presents the top representative BLASTN matches, including GenBank accession numbers, query coverage, percentage identity, and E-values, supporting the molecular identification of the isolate.
